# Prevalence of Common Mental Disorder and Its Association with Khat Chewing among Ethiopian College Students: A Systematic Review and Meta-Analysis

**DOI:** 10.1155/2020/1462141

**Published:** 2020-01-03

**Authors:** Birhanie Mekuriaw, Abriham Zegeye, Alemayehu Molla, Robel Hussen, Solomon Yimer, Zelalem Belayneh

**Affiliations:** ^1^Department of Psychiatry, College of Health and Medical Science, Dilla University, Dilla, Ethiopia; ^2^Department of Biomedical Science, School of Medicine, Debre Markos University, Debre Markos, Ethiopia; ^3^Department of Public Health, College of Health and Medical Science, Dilla University, Dilla, Ethiopia

## Abstract

**Background:**

Mental disorder is a global burden that affects all groups of people. Young people, particularly college/university students, are more vulnerable to common mental disorders than the general population. Thus, students may use khat to gain immediate relief from their psychological distress which may worsen again after longer time of chewing. In Ethiopia, there are studies showing discrepant and inconsistent results of common mental disorder among college/university students. Therefore, this review sought to determine the prevalence of common mental disorder and its association with khat chewing among Ethiopian college/university students.

**Methods:**

Different kinds of literature were searched from the databases of Google Scholar, PubMed/Medline, ScienceDirect, and PsycINFO. A total of 10 primary studies which report the prevalence of common mental disorder among Ethiopian college/university students were included in the review. The data were extracted using a standardized data extraction format prepared in Microsoft Excel. STATA version 14 statistical software was used for analysis. Cochran's *Q* test statistics and *I*^2^ test were used to assess heterogeneity. A random effects meta-analysis model was used to estimate the pooled prevalence of common mental disorder due to the variability of the studies. Lastly, the association between common mental disorder and khat chewing was conducted.

**Results:**

The pooled prevalence of common mental disorder among Ethiopian college/university students was 37.73% (95% CI: 30.43, 45.03). The subgroup analysis showed the highest [51.9% (95% CI: 30.19, 73.70)] and lowest [33.28% (95% CI: 19.95, 46.60)] prevalences of common mental disorder among Ethiopian college/university students found in Amhara and South Nation Nationality and People regions, respectively. The pooled effect (odds ratio) of khat chewing on common mental disorder was 2.01 (95% CI: 1.38, 2.95).

**Conclusions:**

In our review, it is found that more than one-third of college/university students suffered from common mental disorder. Khat chewers were found to be twofolds more vulnerable to develop common mental disorder than nonchewers.

## 1. Background

Common mental disorder (CMD) is a gross name of physical, mental, and social disturbances used to describe a range of symptoms like depression, anxiety, or somatic manifestations which can result long term effect on human life [1, 2]. In a medical sense, CMD has a wider scope that can exhibit a sort of psychiatric symptoms without actually being ill [1, 3]. Nowadays, common mental disorder is a public health problem in which evidences reveled its occurrence among young age groups, particularly college students across the globe [4, 5].

There is an increased alarming rate of khat chewing among college students especially in developing countries which khat has a bimodal relationship with a common mental disorder [6, 7]. Khat (*Catha edulis*) is a psychoactive substance which includes amphetamine like structures and other chemicals [8]. Khat is an ever green plant commonly cultivated in Ethiopia, most East African, and some Asian countries. People initiate khat chewing for its euphoric effect, social engagement, better concentration, some religious activities, and economical purpose [9, 10]. Khat chewing can cause more addiction, and people are facing difficulty to stop chewing as once they have started. Furthermore, the dopamine activity stimulation effect of khat has a brain rewarding system that might reinforce individuals for further chewing and a combination of other psychoactive substances [11].

College students usually experience stressful life events (family separation, educational stress, and cultural shock) which push them to initiate different psychoactive substances including khat to escape from such stressful situations [12]. The brain rewarding and addictive effects of Khat can further result in physical, mental, social, and emotional distress. Its paradoxical effects evidenced by either chewing or withdrawals may also contribute to develop common mental disorders among chewers [3, 13].

In Ethiopia, approximately one-third of college students chew khat [6]. The most commonly mentioned reasons for the initiation of khat chewing were to stay alert, for better concentration and social engagement, and to increase their academic performance [3, 6]. The available literatures in Ethiopia reported highly variable results of prevalence of common mental disorder among college/university students [14, 15]. Moreover, there were great variation and inconsistency related to the prevalence of common mental disorder and its association with khat chewing across different regions of the country. Therefore, the main aim of this systematic review and meta-analysis was to estimate the pooled prevalence of common mental disorder and its association with khat chewing among college students in Ethiopia. The findings of this meta-analysis will help policy makers and other concerned bodies to design appropriate interventional programmers. The study will also be helpful for further research investigations in relation to khat chewing and common mental disorder.

## 2. Methods

### 2.1. Identification and Study Selection

We followed the methods of Gebrie et al. [6]. Published and unpublished research reports related to the prevalence of common mental disorder and its association with khat chewing among Ethiopian college/university students were considered. Literature searches of relevant studies which are written in English language were identified through Google Scholar, PubMed/MEDLINE, ScienceDirect, and PsycINFO. The searching of the articles was carried out from December 10, 2018, to February 16, 2019. The key terms used to retrieve the studies were (Prevalence OR Magnitude OR Epidemiology) AND (Common mental disorder OR Psychological distress OR Mental distress) AND (Khat chewing OR Khat use OR Khat abuse) AND (Ethiopian college/university). All the literatures available until February 16, 2019, were included in the systematic review and meta-analysis. The systematic review and meta-analysis was carried out in accordance with the Preferred Reporting Items for Systematic reviews and Meta-Analyses (PRISMA) guideline [16] (see supplementary [Supplementary-material supplementary-material-1]).

### 2.2. Eligibility Criteria

Abstracts of the searched results were reviewed based on the mentioned inclusion and exclusion criteria.

#### 2.2.1. Inclusion Criteria

The following are the inclusion criteria:

1. Study area: research articles conducted only in Ethiopian college/university were included
2. Study design: observational studies (cross-sectional, case-control, and cohort studies) with original data reporting the prevalence of common mental disorder were included
3. Language: literatures published in the English language were included
4. Population: any studies conducted among Ethiopian college students were included
5. Publication issue: both published and unpublished articles were searched

#### 2.2.2. Exclusion Criteria

First, the eligibility of the studies was evaluated by reading their titles and then abstracts. Primary studies which were not fully accessed were excluded after reading the titles and abstracts since we were unable to assess the quality of the article in the absence of the full texts. Next to that, papers considered relevant to our review after reading the titles and abstracts were selected and their full texts were evaluated again.

### 2.3. Data Extraction

Two authors (BM and AM) independently extracted all the necessary data using a standardized data extraction format prepared in Microsoft Excel. For the first objective (magnitude of common mental disorder), the data extraction format included the first author, publication year, region of the college/university, the name of the university, number of sample size, screening tool used, response rate, and prevalence of mental distress. For the second objective (association between khat chewing and common mental disorder), the data extraction format was prepared in the form of a two by two table. Data were extracted from primary studies in which khat chewing was considered a predictor of common mental disorder. Any disagreements between the two authors during data extraction were solved through discussion and double extraction of the inconsistent data together.

### 2.4. Outcome Measurements

Our systematic review and meta-analysis had two main objectives. First, it was to determine the pooled prevalence of common mental disorder among Ethiopian College students. The second objective was to estimate the pooled effects of khat chewing on common mental disorder among Ethiopian college students. The prevalence of CMD was calculated by dividing the total number of students screened positive for common mental disorder to the total number of students included in the study (sample size) and multiplied by one hundred (100). With regard to the association between khat chewing and common mental disorder, the odds ratio was calculated from the primary studies using two by two tables (see supplementary [Supplementary-material supplementary-material-1]).

### 2.5. Quality Assessment

The Newcastle-Ottawa Scale for cross-sectional studies quality assessment was adapted to assess the quality of the studies included in the review and meta-analysis [17]. Two authors independently evaluated the qualities of the original articles using this assessment tool as a guideline. The tool had indicators consisting of three main parts; the first part has five components and assesses the methodological quality of each study; the second section examines the comparability of the studies, and the last part measures the quality of the original articles with respect to their statistical analysis. The quality of each study was evaluated by using these parameters, and articles with medium (fulfilling 50% of quality assessment criteria) and high quality (6 out of 10 scales) were included for analysis. Disagreements of assessors were solved by taking the mean score of their assessment results.

### 2.6. Statistical Procedure

Important data were extracted using a Microsoft Excel format. After extraction, the data were imported to STATA version 14.0 (software) for analysis. The characteristics of original articles were described using texts, table, and forest plot. The standard error of prevalence for each original article was calculated using the binomial distribution formula. Heterogeneity among the reported prevalence of studies was checked by using a heterogeneity *χ*^2^ test and *I*^2^ test [18]. This heterogeneity tests indicated that there was no significant heterogeneity among the studies as evidenced by *I*^2^ = 97.0% and *p* < 0.001. Therefore, a random effects meta-analysis model was used to estimate the Der Simonian and Laird's pooled prevalence of common mental disorder and its association with khat chewing. Publication bias was also examined by performing Egger's correlation and Begg's regression intercept tests at a 5% significant level [19, 20]. The results of these tests indicated that there was no publication bias as evidenced by *p* = 0.273 in Egger's test. Moreover, subgroup analysis was done based on the region of studies conducted and publication year to minimize the random variations between the point estimates of the primary studies.

## 3. Results

### 3.1. Search Results

**Figure 1:**
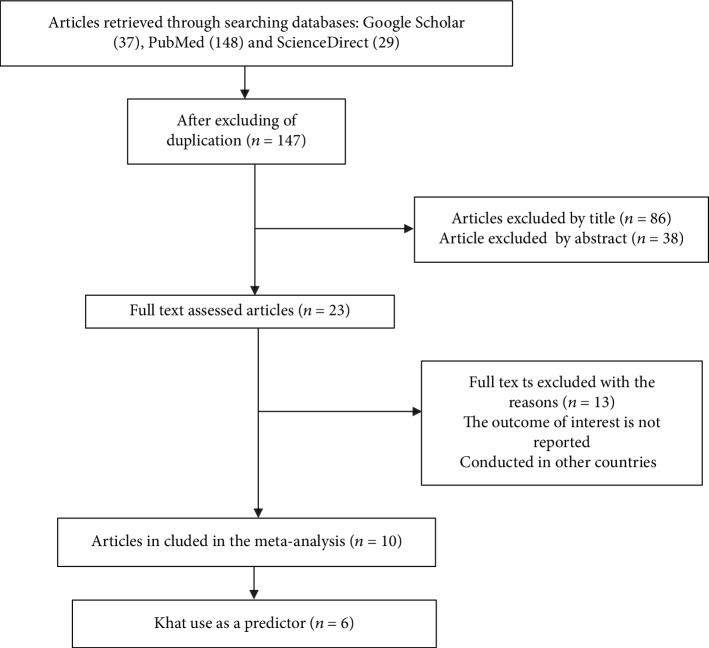
Flow chart explaining the selection of primary studies for the systematic review and meta-analysis of prevalence of common mental disorder and its association with khat chewing among Ethiopian college students (2019).

In the first step of our search, 214 articles were retrieved regarding the prevalence of common mental distress among college/university students using Midline/PubMed, Google Scholar, ScienceDirect, and PsycINFO. From the 214 articles, 67 articles were excluded due to duplication. Additionally, 124 articles were excluded after reviewing their titles and abstracts in which we found them as nonrelevant to our review and inaccessibility of their full text. Hence, we read the full texts of 23 articles and assessed for their eligibility based on the preset criteria. About 13 articles were further excluded due to the differences in the study population and study settings. Among these, six were conducted in Saudi Arabia [21, 22], Pakistan [23], Brazil [24], Egypt [25], and Somalia [26]. The other six studies were excluded because of their different study population (conducted other than college/university students) [27–32]. The remaining one study was also excluded as the study subjects were taken from two universities at the same time (Gondar and Haramya) [33]. Finally, 10 articles were found to be eligible and included in the systematic review and meta-analysis (Figure 1).

### 3.2. Original Article Characteristics

Table 1 shows the summary of the characteristics of ten (10) primary studies included in our systematic review and meta-analysis. The study was conducted from December 10, 2018, to February 16, 2019, in the four regions of the country (two from Amhara region [15, 34], three from Oromia [14, 35, 36], four from SNNPR [37–40], and one from Tigray region [41]) were included. Regarding the assessment tools, one study at Debre Berhan University [15] was done using the Kesler 10-item questionnaire (K-10) while others used a self-reported questionnaire (SRQ) to screen common mental disorder. All the articles included in the review were cross-sectional studies with the sample sizes ranging from 240 to 1198 [39, 40]. The lowest and highest prevalences of common mental disorder were reported from Adama University [14] and Debre Berhan University students [15], respectively.

### 3.3. Meta-Analysis

**Figure 2:**
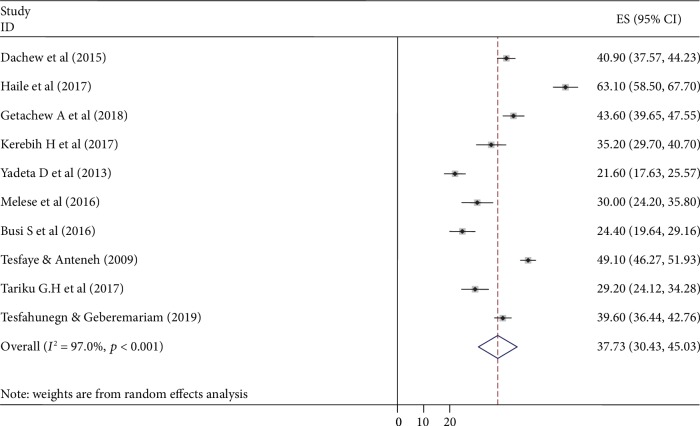
Forest plot for the pooled prevalence of common mental disorder among college students in Ethiopia (2019).

The forest plot shows the results of total 10 primary studies included in our review (Figure 2). The pooled prevalence of common mental disorder among Ethiopian collage/university students was 37.73% (95% CI: 30.43, 45.03). Heterogeneity was seen across the studies which is uncovered by *I*^2^ statistic (*I*^2^ = 97.0%, *p* value *<* 0.001). Therefore, a random effects model was conducted to estimate the pooled prevalence of mental distress among college students in Ethiopia. With regard to publication bias, Begg's and Eggers's tests were checked and no significant publication bias observed as evidenced by *p* = 0.371 and *p* = 0.273, respectively.

### 3.4. Subgroup Analysis

In our meta-analysis, we performed a subgroup analysis based on the regions where the studies were conducted and publication years. From the four regions, the lowest prevalence [33.28% (95% CI: 19.95, 46.60)] of common mental disorder was found in SNNPR whereas the highest prevalence [51.94%, (95% CI: 30.19-73.70%)] of common mental disorder was indicated in Amhara region. Regarding publication year, the prevalence of common mental disorder was higher [42.18 (95% CI: 31.91, 52.45)] among studies done after 2016 than studies done at 2016 and before [33.29 (95% CI: 22.09, 44.49)] (Table 2).

### 3.5. Association of Common Mental Disorder and Khat Chewing

**Figure 3:**
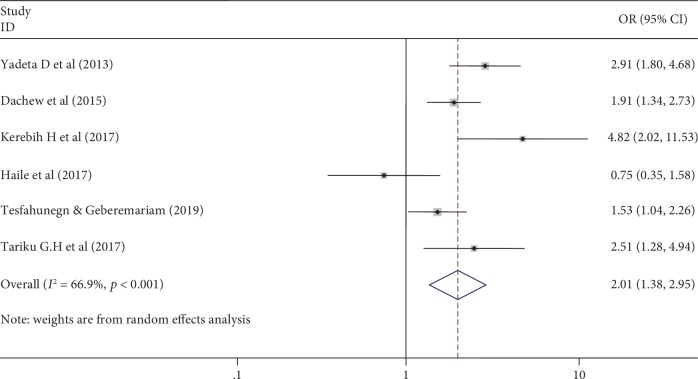
Forest plot showing pooled odds ratio of khat chewing in association with common mental disorder among Ethiopian college/university students (2019).

Khat chewing was considered a predictor of common mental disorder among sixty percent (60%) of the primary studies included in our systematic review and meta-analysis. Accordingly, the pooled odds ratio of khat chewing showed that khat chewers were two times more vulnerable to develop common mental disorder than their counterparts (Figure 3).

## 4. Discussion

Substance abuse is substantially increasing among college/university students across the globe due to the academic-related stresses and many others reasons [6, 42]. Recently, different studies indicate that khat is the commonly abused psychoactive substance in most all Ethiopian college or university students [6]. This is strongly associated with a huge physical, psychological, social, and interpersonal problems for the individuals engaged in chewing and the community at large [6, 43]. However, the results of such studies have highly variable results in Ethiopia ranging from 21.6% to 63.1%. Therefore, determining the pooled prevalence and cumulative quantification of the risk of khat chewing for common mental disorder among college students has a paramount importance.

In this systematic review and meta-analysis, we investigated the prevalence of common mental disorder as well as its association with khat chewing across 10 different primary studies comprising a total of 5318 college/university students.

The pooled prevalence of common mental disorder among college students was 37.73 (95% CI: 30.43, 45.03). This finding is in line with studies done among university students of Iran (33%) [44] and Brazil (31.5%) [45].

Our review finding showed a higher prevalence of common mental disorder as compared to the WHO estimate of common mental disorder among the general population in African regions (8%) [1]. This might be explained by the fact that college and university students face different academic-related and psychosocial stresses that can expose them to develop common mental disorders. College/university life is the time at which students try to live out of their family members and try to socialize in a new environment with people from different religions, ethnicities, and cultural contexts. This might create a cultural shock with a possible risk for developing common mental disorder. Beyond to this, students commonly join colleges and universities at the time of their puberty age in which their hormonal and psychological makeup is progressed. In addition, this difference can be explained by differences in the number of study participants included, difference in the study periods of the studies, and differences in the segment of population included.

The subgroup analysis of this review showed that the prevalence of common mental disorder s was found to be higher [42.18% (95% CI: 31.91, 52.45)] among the studies conducted after 2016 than studies done at 2016 and before [33.29% (95% CI: 22.09, 44.49)]. This might be due to the contemporary consideration of khat chewing as a characteristic of modern life fashion follower among youngsters.

The association between Khat chewing and common mental disorder was computed among six primary studies from the total of 10 articles included in this review. Hence, khat chewing increases the risk of having common mental disorder by twofolds among college/university students. This might be explained by the direct brain effect of Khat and the physical mental and social crisis of chewing after long term use. Despite khat chewing being considered the contemporary fashion and a characteristic of modern life among youngsters, it is totally prohibited in all religions and elders in Ethiopia. Individuals who use khat commonly segregate from their former religious and parental social bonds. This can contribute its effect for the development of common mental disorder. There is also an amazing practice among khat chewers in Ethiopia; people drink alcohol over night after chewing khat to overcome the euphoric effect of khat and to calm down their hyperactivity as a self-medication [42]. This long-term alcohol and khat consumption might be a significant risk for common mental disorder.

## 5. Conclusions

There is a high burden of common mental disorder among Ethiopian college students. Common mental disorders lead to considerable losses in health and functioning which can be quantified at the population level by estimates of years lived with disability (YLD). Globally, depressive disorders and anxiety disorders are responsible for 74.6 million years lived with disability (YLD) among the general population in 2015. However, the global prevalence of common mental disorder was 8% in 2015 [1]. It can be easily understood that the high prevalence (37.7%) of CMD among Ethiopian students poses a great risk of years lived with disability which could hinder the learning and creative potentials of the students. It can also have a great impact on the country's future economic, social, and overall developments. Although lots of attempts have been made to reduce the highly prevalent CMD among youths and students, a more holistic and integrated effort is required to minimize its burden and future impacts. Intervening drug abuse like khat chewing is also highly recommended.

## Figures and Tables

**Table 1:** Summary table for the prevalence of common mental disorder among 10 studies of Ethiopian college students included in the systematic review and meta-analysis (2019).

Regions	Colleges	First author	Publication year	Sample size	Assessment tools	Response rate	Quality assessment	Prevalence (95% CI)
Amhara	Debre Berhan	Haile et al. [15]	2017	422	K-10	100	7	63.10 (58.50, 67.70)
	Gondar	Dachew et al. [34]	2015	836	SRQ-20	95.8	9	40.90 (37.57, 44.23)
SNNRP	Hawassa	Melese et al. [39]	2016	240	SRQ-20	100	8	30.00 (24.20, 35.80)
	Hawassa	Busi et al. [38]	2016	327	SRQ-24	95.4	6	24.40 (19.64, 29.16)
	Hawassa	Tesfaye [40]	2009	1198	SRQ-20	99.5	8	49.10 (46.27, 51.93)
	Mizan Aman	Jini [37]	2017	308	SRQ-20	97.2	7	29.20 (24.12, 34.28)
Oromia	Adama	Dessie et al. [14]	2013	413	SRQ-20	95.3	7	21.60 (17.63, 25.57)
	Jimma	Kerebih et al. [35]	2017	290	SRQ-20	95	7	35.20 (29.70, 40.70)
	Mada Wolabu	Getachew and Tekle [36]	2018	605	SRQ-20	100	6	43.60 (39.65, 47.55)
Tigray	Aksum	Tesfahunegn and Gebremaraim [41]	2019	919	SRQ-20	95.1	8	39.60 (36.44, 42.76)

**Table 2:** Subgroup prevalence of common mental disorder among Ethiopian college students (2019) (*n* = 10).

Variables	Characteristics	Number of studies	Prevalence rate (95% CI)
Region	Amhara	2	51.94 (30.19, 73.70)
	Oromia	3	33.45 (19.53, 47.38)
	SNNPR	4	33.28 (19.95, 46.60)
	Tigray	1	39.60 (36.44, 42.76)
Publication year	≤2016	5	33.29 (22.09, 44.49)
	>2016	5	42.18 (31.91, 52.45)
Overall		10	37.73 (30.43, 45.03)

## Abbreviations

CMD: Common mental disorder
SNNPR: Southern Nations, Nationalities, and People's Region
SRQ: Self-reported questionnaire
WHO: World Health Organization
YLD: Years lived with disability

## Supplementary Materials

Supplementary 1 PRISMA-P (Preferred Reporting Items for Systematic review and Meta-Analysis Protocols) 2015 checklist: recommended items to address in a systematic review protocol.

Supplementary 2 Two by two table showing a data extraction format to calculate the association between common mental disorder and khat chewing.
